# NMR determination of the 2:1 binding complex of naphthyridine carbamate dimer (NCD) and CGG/CGG triad in double-stranded DNA

**DOI:** 10.1093/nar/gkac740

**Published:** 2022-09-12

**Authors:** Takeshi Yamada, Kyoko Furuita, Shuhei Sakurabayashi, Makoto Nomura, Chojiro Kojima, Kazuhiko Nakatani

**Affiliations:** Department of Regulatory Bioorganic Chemistry, SANKEN, Osaka University, 8-1 Mihogaoka, Ibaraki 567-0047, Japan; Institute for Protein Research, Osaka University, 3-2 Yamadaoka, Suita, Osaka 565-0871, Japan; Department of Regulatory Bioorganic Chemistry, SANKEN, Osaka University, 8-1 Mihogaoka, Ibaraki 567-0047, Japan; Institute for Protein Research, Osaka University, 3-2 Yamadaoka, Suita, Osaka 565-0871, Japan; Graduate School of Biological Sciences, Nara Institute of Science and Technology, 8916-5 Takayama, Ikoma 630-0192, Japan; Institute for Protein Research, Osaka University, 3-2 Yamadaoka, Suita, Osaka 565-0871, Japan; Graduate School of Engineering Science, Yokohama National University, 79-5 Tokiwadai, Hodogaya-ku, Yokohama 240-8501, Japan; Department of Regulatory Bioorganic Chemistry, SANKEN, Osaka University, 8-1 Mihogaoka, Ibaraki 567-0047, Japan

## Abstract

Trinucleotide repeat (TNR) diseases are caused by the aberrant expansion of CXG (X = C, A, G and T) sequences in genomes. We have reported two small molecules binding to TNR, **NCD**, and **NA**, which strongly bind to CGG repeat (responsible sequence of fragile X syndrome) and CAG repeat (Huntington's disease). The NMR structure of **NA** binding to the CAG/CAG triad has been clarified, but the structure of **NCD** bound to the CGG/CGG triad remained to be addressed. We here report the structural determination of the **NCD**-CGG/CGG complex by NMR spectroscopy and the comparison with the **NA**-CAG/CAG complex. While the **NCD**-CGG/CGG structure shares the binding characteristics with that of the **NA**-CAG/CAG complex, a significant difference was found in the overall structure caused by the structural fluctuation at the ligand-bound site. The **NCD**-CGG/CGG complex was suggested in the equilibrium between stacked and kinked structures, although **NA**-CAG/CAG complex has only the stacked structures. The dynamic fluctuation of the **NCD**-CGG/CGG structure at the **NCD**-binding site suggested room for optimization in the linker structure of **NCD** to gain improved affinity to the CGG/CGG triad.

## INTRODUCTION

**Figure 1.**
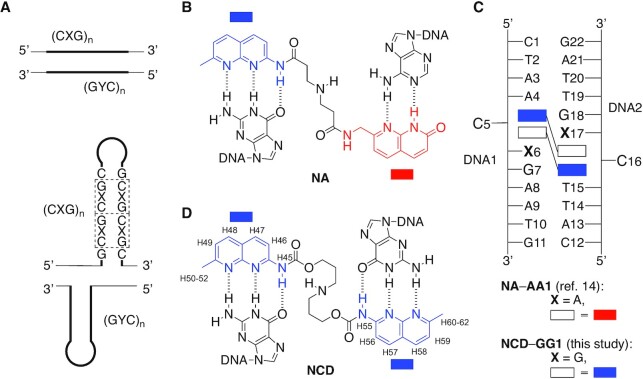
(**A**) Illustration of the slip-out structure of (CXG)n/(CYG)n repeat in the double-stranded DNA. Chemical structures of (**B**) **NA** and (**D**) **NCD**. The proton numbering scheme of naphthyridine moiety in **NCD** is also shown along with the chemical structure. (**C**) Illustration of the **NA**-**AA1** complex comprised of two **NA** molecules and a dsDNA containing a CAG/CAG triad DNA (**AA1**) and the **NCD**-**GG1** complex (this study) comprised of two **NCD** molecules and a dsDNA containing a CGG/CGG triad DNA (**GG1**). Rectangle shapes with red and blue color are azaquinolone (AQ) and naphthyridine (NP) heterocycle, respectively.

The aberrant expansion of trinucleotide repeat (CXG, X = A, C, G and T) sequences causes more than 40 neurological disorders. The CGG expansion in the *FMR1* gene causes the neurological disorder fragile X syndrome (1–5). Healthy individuals have the CGG repeat length from 6 to 50, whereas the fragile X syndrome patients with the full mutation have more than 200 repeats (6). Like fragile X syndrome, the aberrant expansion of CXG repeat causes Huntington's disease (HD, CAG repeat), spinocerebellar ataxia type 12 (SCA12, CAG), myotonic dystrophy type 1 (DM1, CTG), SCA8 (CTG), and Friedreich ataxia involving non-CXG repeat GAA (7–11). In trinucleotide repeat diseases, the expanded repeats form metastable slip-outs consisting of a hairpin structure with a repeated unit of the CXG/CXG triad motif, where two C-G base pairs flank X–X mismatches (Figure 1A, bottom panel). The CGG repeats in the genome have fully complementary CCG repeats in the opposite strand, and each strand can form slip-out structures in the aberrantly expanded state during the biological reactions such as replication and transcription, where the dissociation of the duplex is involved. The chemical stability of the slip-out is one of the crucial factors determining the genomic repeat instability leading to the repeat expansion and contraction. The longer the repeat length, the higher propensity in forming slip-out structures is conceivable (12). We could anticipate that the intervention of the slip-out structures by small external molecules (i.e. ligands) could modulate the chemical stability of the hairpin structure, and hence the genomic repeat instability.

In 2020, we reported that a small molecule naphthyridine-azaquinolone (**NA**) (13,14) (Figure 1B) binding to the CAG/CAG triad motif in the slip-out produced on the CAG repeat (Figure 1A, X = A and Y = T) induced the repeat contraction in the striatum of the R6/2 mouse model of Huntington's diseases (15). Huntington's disease is caused by the aberrant expansion of the CAG repeat in the *HTT* gene in chromosome 4. **NA** was originally designed and developed with the anticipation of the binding to the G-A mismatch, as two heterocycles, 2-amino-1,8-naphthyridine (NP, colored in blue in Figure 1B) and 8-azaquinolone (AQ, colored in red in Figure 1B), composing **NA** can form hydrogen bonds with guanine and adenine bases, respectively. The unexpected **NA** binding to the CAG/CAG motif was found by the increased thermal stability of the duplex DNA containing a CAG/CAG triad in the presence of **NA**. The characteristic 2:1 binding stoichiometry of **NA** to the duplex containing CAG/CAG motif was determined by cold spray ionization time-of-flight (CSI-TOF) mass spectrometry and isothermal titration calorimetry (ITC), as well as NMR titration experiments. NMR structural analysis of the complex of **NA** bound to a CAG/CAG triad DNA (**AA1**, Figure 1C) revealed the simultaneous binding of two **NA** molecules to the triad, the formation of hydrogen bonding between AQ-adenine and NP-guanine, and the cytosine flipping-out from the base stacks (14). **NA** binding to the CAG repeat DNA was confirmed by SPR analysis with the sensor chip carrying CAG repeat DNA on the surface. CSI-TOF mass spectrometry of (CAG)n repeat DNA and **NA** provided ions of complexes with an even number of **NA**, suggesting the formation of 2:1 complex on the CAG repeat slip-out (14). A structure-binding activity relationship study on the linker connecting two heterocycles revealed that the **NA** binding to the CAG/CAG triad motif is sensitive to the chemical structure of the linker (16).

The remarkable finding that the CAG repeat binding molecule **NA** induced the repeat contraction *in vivo* prompted us to reinvestigate the small molecules binding to the CXG/CXG triad in detail from the structural viewpoint to improve the binding affinity. Various ligands binding to the mismatches have been reported to date (13,14,17–39). Our group has developed a series of mismatch binding ligands, including **NA** and naphthyridine carbamate dimer **NCD** (17,18) (Figure 1D). **NCD**, which consists of two NPs and a three-methylene linker connecting them by a carbamate linker, was developed based on the first-generation molecule binding to the G-G mismatch (23). **NCD** was found to bind to the CGG/CGG triad motif with the stoichiometry of 2:1 as determined by CSI-TOF MS. Two cytosines in the **NCD**-CGG/CGG complex were suggested in the flipped-out position by hydroxylamine probing reaction (18,40,41). While these binding features of **NCD** to the CGG/CGG motif were similar to those observed for the **NA** binding to the CAG/CAG motif, the structure of **NCD** bound to the CGG/CGG motif DNA was remained to be addressed for 15 years after its discovery. The proposed mechanism of **NA**-induced repeat contraction involves the escape of **NA**-bound CAG hairpin produced during the transcription from the repair processes. While the precise mechanics need further studies, from the viewpoint of chemical and structural biology research, the relevance between structures of **NA**-CAG/CAG and **NCD**-CGG/CGG could be a clue in developing the small molecules contributing to the repeat contraction.

We here report the structure determination of **NCD** bound to the CGG/CGG triad motif DNA, confirming the 2:1 binding stoichiometry, formation of four NP-G hydrogen-bonded pairs, and flip-out of cytosines. We used dsDNA containing the CGG/CGG triad in the middle (**GG1**, Figure 1C) as a model of the CGG/CGG triad in the CGG slip-out hairpin. The determined structure of **NCD** bound to the CGG/CGG triad indicated the possible kink at the step between G6-NP and G17-NP pairs (residue numbers are shown in Figure 1D), highlighting the difference from the **NA**-CAG/CAG structure, and most importantly, suggesting the possibility for further optimization of the linker structure of **NCD** to improve the affinity to the CGG/CGG triad.

## MATERIALS AND METHODS

### Sample preparation for NMR measurements

The chemically synthesized DNA oligomers 5′-d(CTAA CGG AATG)-3′ and 5′-d(CATT CGG TTAG)-3′ were purchased from commercial suppliers (FASMAC, GeneDesign, and Hokkaido system science). Each oligomer was dissolved in 20 mM sodium phosphate buffer (pH 6.8) containing 100 mM NaCl at a concentration of about 6 mM. The DNA solution of each strand was mixed at a molar ratio of 1:1 and annealed overnight. To remove anionic impurities, the obtained double-stranded DNA (dsDNA) solution was dialyzed three times using a microdialysis cup (molecular weight cut-off 3,500) (Bio-Tech International, Inc.) against 20 mM sodium phosphate buffer (pH 6.8) containing 1 M NaCl. Then, the dsDNA solution was dialyzed three times against 20 mM sodium phosphate buffer (pH 6.8) containing 100 mM NaCl. **NCD** was synthesized according to the previously reported protocol (42). **NCD** (3.35 mg) was dissolved in 50 μl of 20 mM sodium phosphate buffer (pH 6.8) containing 100 mM NaCl. The final concentration of **NCD** was 125 mM and confirmed by UV absorbance at 321 and 332 nm.

### NMR measurements

Titration experiments were carried out at 283 K using 1D 1–1 echo ^1^H measurements. The dsDNA was prepared at a concentration of 50 μM in 90/10% H_2_O/D_2_O containing 20 mM sodium phosphate (pH 6.8) and 100 mM NaCl. For resonance assignments and structure calculations of **NCD**-**GG1**, the NMR sample of **NCD**-**GG1** was prepared at a concentration of 2.5 mM in 90/10% H_2_O/D_2_O containing 20 mM sodium phosphate (pH 6.8) and 100 mM NaCl. **NCD** was added to the DNA solution in molar ratios of 1:0.4, 1:0.8, 1:1.2, 1:1.6 and 1:2.0. Using this sample, ^1^H–^1^H NOESY with mixing times of 30 and 200 ms, ^1^H–^1^H TOCSY, DQF-COSY, and ^1^H–^31^P HSQC spectra were measured on a Bruker DRX800 spectrometer, and ^1^H–^13^C HSQC spectra were measured on a Bruker AVANCE500 spectrometer.

### Structure determination

NOE distance restraints were obtained from a ^1^H–^1^H NOESY spectrum with a mixing time of 200 msec, and a recycle delay of 7.3 s. Cross peaks in the spectrum were integrated using NMRFAM-SPARKY (43). Interproton distance restraints were determined from the integrated peak intensities by the random error MARDIGRAS (RAND MARDI) procedure of the complete relaxation matrix analysis method (44). Based on DQF-COSY, NOESY and ^1^H–^31^P HSQC spectra, sugar puckers and backbone torsion angles were restrained to maintain an S-type sugar conformation and right-handed helix, respectively. Hydrogen bonding restraints were imposed on the Watson-Crick base pairs and the NP-guanine pairs. After all, 358 distance constraints including 56 intermolecular NOE distances and 180 dihedral angle constraints were collected. With these constraints, a total of the 300 complex structures was calculated using a simulated annealing protocol using Crystallography & NMR System (CNS) version 1.3 (45). Thirty structures without a distance violation >0.5 Å were selected.

### RDC measurements

For residual dipolar coupling (RDC) measurements, the two NMR samples of **NCD**-**GG1** were prepared at a concentration of 2.5 mM in 100% D_2_O containing 20 mM sodium phosphate (pH 6.8) and 100 mM NaCl, one of which contained 20 mg/ml Pf1 phage (ASLA BIOTECH AB), and the other did not. Using these samples, DQF-COSY spectra were measured on the Bruker DRX800 spectrometer, and ^1^H–^13^C IPAP HSQC spectra were measured on the Bruker AVANCE500 spectrometer. All measurements were carried out at 283 K. The alignment was confirmed by quadrupolar splits of ^2^H NMR signals (12 Hz at 500 MHz ^1^H frequency).

### RDC analyses

Sixteen ^13^C–^1^H RDC (D_CH_) values were obtained from the ^1^H–^13^C IPAP HSQC spectra using SPARKY. Eight ^1^H–^1^H RDC (D_HH_) values were obtained from the DQF-COSY spectra using amplitude-constrained multiplet evaluation (ACME) software (46). The correlation coefficient r between these experimental RDCs and the RDCs back-calculated from the NMR structure was calculated using PALES software (47).

## RESULTS

### Binding assay

Prior to the NMR experiments, we performed binding assays on the **NCD**-CGG/CGG complex using the dsDNA **GG1** comprised of DNA1: 5′-d(CTAA CGG AATG)-3′ and DNA2: 5′-d(CATT CGG TTAG)-3′. **GG1** DNA is also used for NMR experiments. In brief, the UV absorbance changes at 260 nm of **GG1** (5 μM) showed the typical sigmoidal curve with the melting temperature (*T*_m_) value of 26.8°C (Supplementary Figure S1A in ESI). The *T*_m_ value of **GG1** increased to 48.6°C in the presence of 20 μM **NCD**. The CD spectrum of **GG1** in the presence of **NCD** showed the induced CD bands around 350 nm of the **NCD** absorption range, indicating that non-chiral molecule **NCD** is located in a chiral environment of **GG1** (Supplementary Figure S1B). Binding stoichiometry was determined by CSI-TOF MS with the hairpin DNA consisting of **GG1** with a T4 hairpin loop. The observed ions at 1797.8 (calcd. 1797.5) and at 1497.9 (calcd: 1497.8) were found to correspond to the 5^–^ and 6^–^ ions of 2:1 **NCD**-DNA complex, respectively, confirming the 2:1 binding stoichiometry. The 1:1 complex of **NCD**:DNA was not observed under the conditions. ITC measurements provided the apparent *K*_D_ of 67 nM with Δ*G* of –9.79 kcal/mol, Δ*H* of –29.5 kcal/mol and Δ*S* of –66.1 cal/mol/deg (Supplementary Figure S1D, below). The binding stoichiometry calculated by the ITC data was 2.06, which was in good agreement with the results of CSI-TOF MS experiments. All the data confirmed our previously reported data (17,18).

### NMR titration experiment

**Figure 2.**
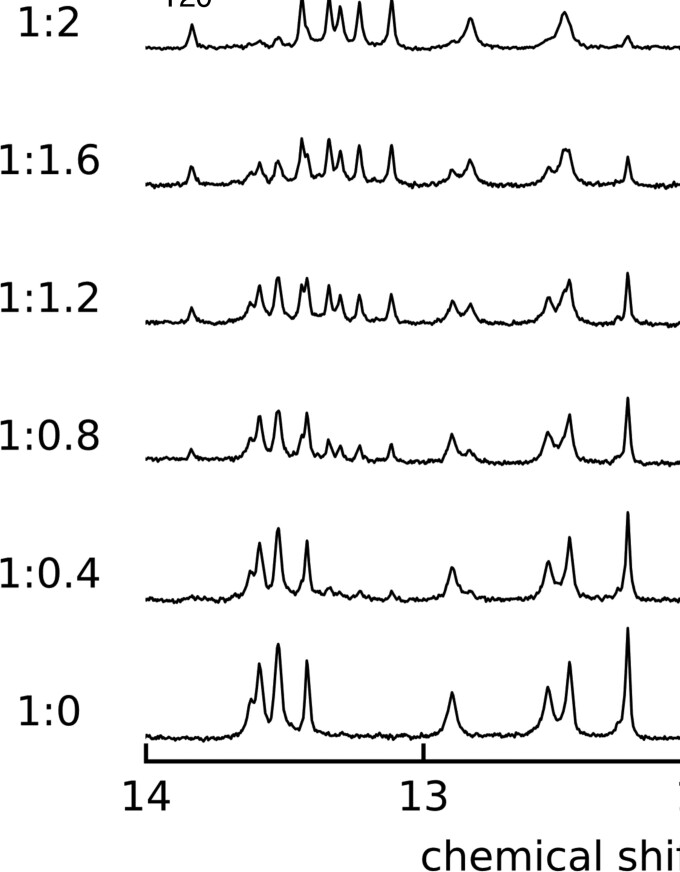
One-dimensional ^1^H spectra of **GG1** (50 μM) in sodium phosphate buffer (20 mM, pH 6.8) containing 100 mM NaCl in the presence of **NCD** with various **GG1**-**NCD** ratios at 283 K. The molar ratio of **GG1** and **NCD** was shown in the left (**GG1**:**NCD**). NC-NH indicates the amide protons of **NCD**.

The 1D ^1^H-NMR titration was performed to investigate the features of **NCD** binding to the CGG/CGG triad motif. The signals of **GG1** DNA from 10.5 to 14 ppm were monitored at 283 K (Figure 2). The *T*_m_ value of **GG1** of 26.8°C (299.9 K) indicated that most of **GG1** exists as a duplex form at 283 K. As the amount of **NCD** increased, proton signals observed between 12 and 14 ppm decreased the intensity without changing chemical shifts and peak shapes with the concomitant appearance of eight new peaks between 10.5 and 12.0 ppm. These new signals were identified as imino protons of G6, G7, G17 and G18 in the CGG/CGG triad and amide protons of **NCD**. These behaviors of proton signals suggested a slow exchange between the free **GG1** and the **NCD**-bound state within the NMR time scale. The spectral changes by **NCD** titration reached saturation at the 2:1 ratio of **NCD** and **GG1**, showing a good agreement with the results of CSI-TOF MS (Supplementary Figure S1C) and ITC (Supplementary Figure S1D). In addition, the binding of two **NCD** molecules to **GG1** producing a 2:1 **NCD**-**GG1** complex was found highly cooperative as any signals corresponding to the intermediates such as the 1:1 **NCD**-**GG1** complex were not observed under the conditions.

### ^1^H and ^31^P assignments of the NCD-GG1 complex

**Figure 3.**
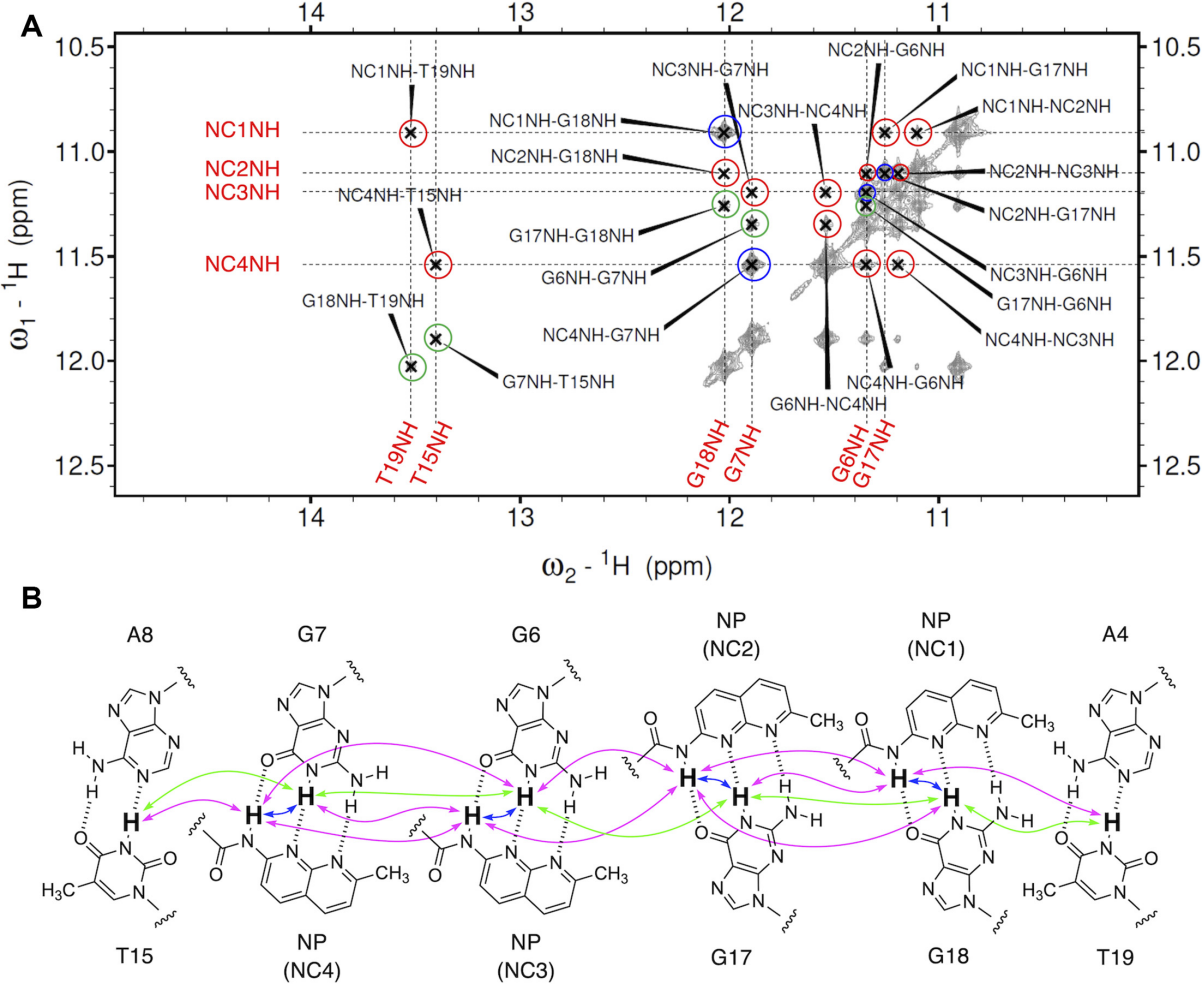
(**A**) ^1^H–^1^H NOESY spectrum of the **NCD**-**GG1** complex. Imino proton region (10–15 ppm) is shown expanded. Intermolecular NOEs between imino protons (G18, G17, G6 and G7) of guanine and amide protons of NPs (NC1 to NC4) within a base pair are indicated by blue circles. Sequential NOEs between imino and amide protons and between amide protons are indicated by red circles. Sequential NOEs between imino protons are indicated by green circles. (**B**) The schematic illustration shows the binding of two **NCD** molecules to the binding site. Blue, red, and green arrows correspond to the blue, red and green circles in Figure 3A.

The NOESY spectrum of the imino proton region observed in 10% D_2_O is shown in Figure 3A. Imino protons were assigned by the imino–imino sequential NOEs starting from T2 to T10 (Figure 3B, green arrows, the region from A4 to A8 was shown for clarity). Sequential NOEs between NH amide protons in **NCD** (indicated by NCnNH, where n is 1, 2, 3 and 4) and base imino protons (Figure 3B, magenta arrows) were also identified. The strong NOESY cross peaks observed between NC1NH and G18NH, NC2NH and G17NH, NC3NH and G6NH, and NC4NH and G7NH (Figure 3B, blue arrows) indicate that the imino protons of guanine and amide protons of **NCD** in each pair are in close vicinity, suggesting the formation of four hydrogen-bonded pairs of NC1-G18, NC2-G17, NC3-G6 and NC4-G7. The NOESY cross peaks of **NCD**-**GG1** were sequentially assigned by identifying intra- and internucleotide NOEs between aromatic H6/8 and sugar H1’ protons. The sequential assignment was achieved throughout **NCD**-**GG1**, including the CGG/CGG region (Supplementary Figure S2). Intensities of all cross peaks in NOESY were consistent with a B-form DNA (48). The H5 and H6 of cytosines and aromatic protons of NP moieties of **NCD** were assigned by ^1^H-^1^H TOCSY (Supplementary Figure S3) and DQF-COSY (Supplementary Figure S4) spectra. The observation of NOE contacts between the aromatic moieties of **NCD** and **GG1** provides evidence of the insertion of **NCD** into base stacks of DNA. The ^1^H–^31^P HSQC spectra showed that ^31^P chemical shifts of **GG1** were distributed from –4.5 to –4.0 ppm and –4.7 to –2.8 ppm in the absence and presence of **NCD**, respectively. The large downfield shifts of ^31^P signals were observed at the 3′-side of the CGG triad (G7 and G18) (Supplementary Figure S5).

### NMR structure of the NCD-GG1 complex

**Figure 4.**
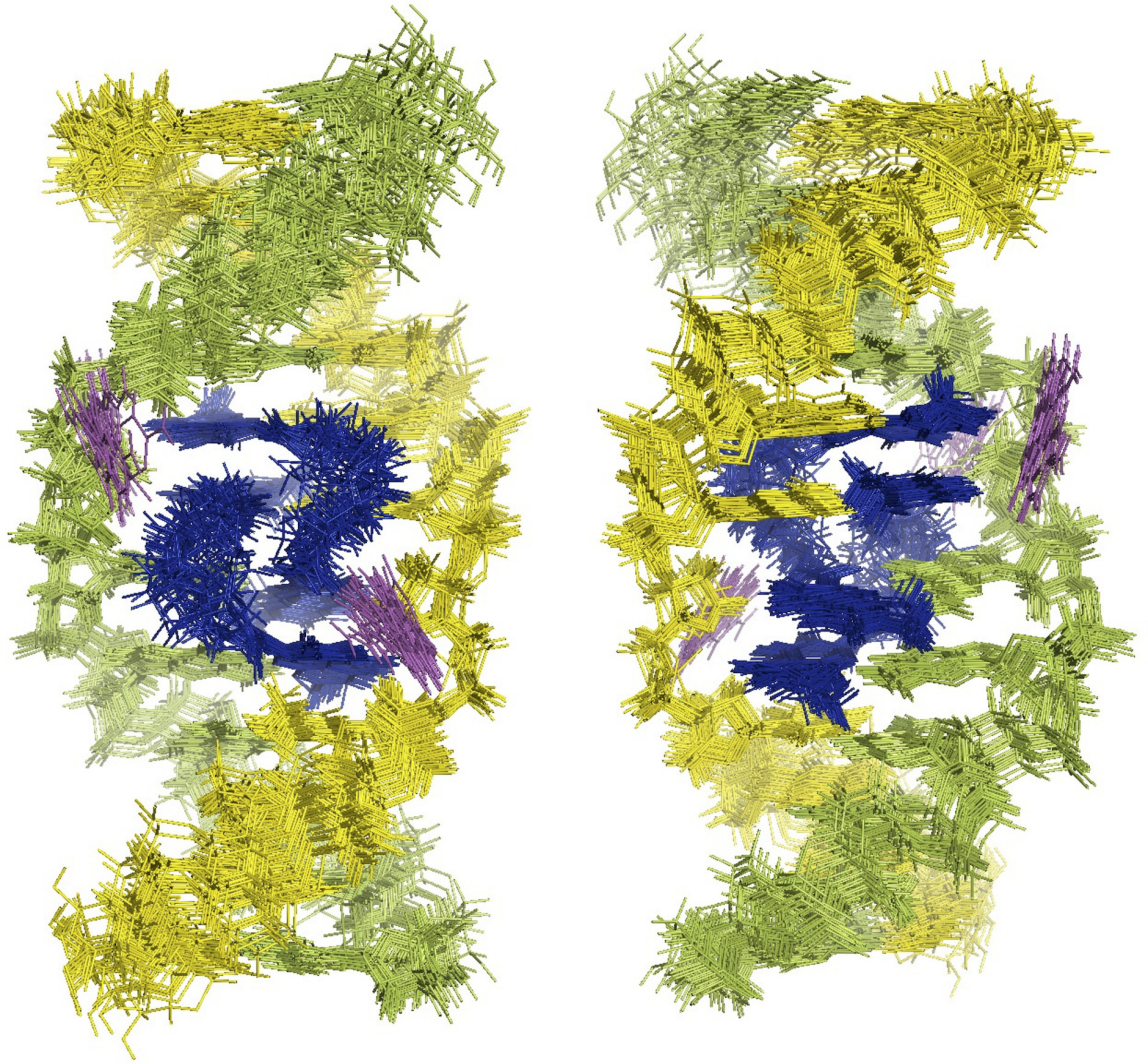
Superimposed representations of the 30 lowest-energy NMR structures of the **NCD**-**GG1** complex. Two NCD molecules are colored in blue. DNA strands are colored in yellow and green. The flipped-out cytosine bases are colored in magenta. The structures are seen from the major groove (left) and minor groove (right).

**Figure 5.**
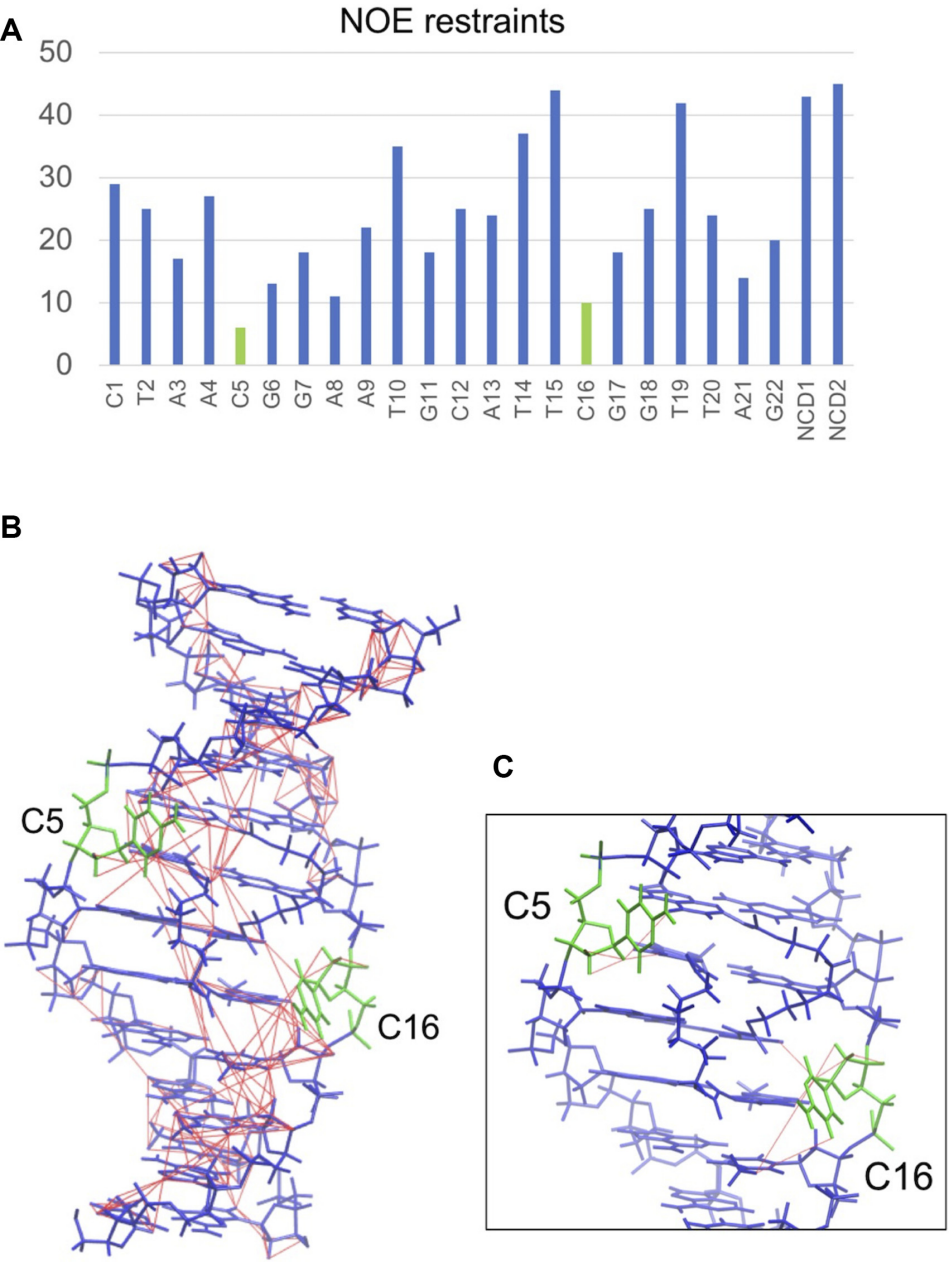
(**A**) The number of NOE restraints for each residue. (**B**) NOE restraints (thin red lines, drawn by VMD-XPLOR (Schwieters, C.D. & Clore, G.M. The VMD–XPLOR visualization package for NMR structure refinement. *J. Magn. Reson*. 149, 239–244 (2001).)) used for the structure determination are shown on the lowest energy structure of the **NCD**-**GG1**. C5 and C16 are shown in green, and the other nucleotides and **NCD** molecules are shown in blue. (**C**) An enlarged view near C5 and C16 is shown; only NOEs containing C5 or C16 are shown.

Superimpose of the 30 lowest-energy NMR structures (state 1–30) of the **NCD**-**GG1** is shown in Figure 4, and the structural statics are summarized in Supplementary Table S1. The number of NOE restraints used for the calculation is shown as a graph in Figure 5A. The number of NOE signals associated with C5 and C16 was much lower than those with other residues, indicating that these cytosine bases are likely out of the DNA base stacking. The torsion angle constraints used for the **NCD**-**GG1** are the same as those used for the calculation of the **NA**-**AA1** structure. The NOE restraints used for the calculation are shown on the lowest energy structure (state 1) of **NCD**-**GG1** (Figure 5B). The distance restraints associated with C5 and C16 are shown in Figure 5C.

## DISCUSSION

We used the CGG/CGG triad in **GG1** as a model of the CGG/CGG triad in the CGG slip-out hairpin. The structure of the CGG/CGG triad embedded in the double-stranded DNA would be different from the CGG/CGG triad produced in the CGG slip-out hairpin. However, there are some relevancies in the binding characteristics between the NCD binding to the CGG/CGG in **GG1** and CGG repeat DNA. CSI-TOF MS of d(CGG)_10_ in the presence of **NCD** produces the significantly strong ion peaks corresponding to [CGG_10_ + 4**NCD**]^6–^ and [CGG_10_ + 6**NCD**]^6–^, containing an even number of **NCD** molecules, while a peak corresponding to [CGG_10_ + 5**NCD**]^6–^ has a comparably small intensity (Supplementary Figure S6). This suggests that two sets of **NCD** molecules favorably bind to the binding site, likely a CGG/CGG triad. The induced CD signal obtained for d(CGG)_10_ with **NCD** is quite similar to that obtained for the **NCD**-**GG1** complex (18). Also, **NCD** significantly increases the *T*_m_ value of d(CGG)_10_ as well as **GG1** (Supplementary Figure S7). These similarities in the binding characteristics showed that the CGG/CGG triad embedded in the dsDNA would be a reasonable model for the triad in the CGG slip-out hairpin. For the comparison of the results of binding analyses (*T*_m_, CD, MS and ITC), we used the **GG1** DNA for all NMR experiments. One dimensional ^1^H spectra of **GG1** titrated by **NCD** clearly indicated (a) the **GG1** signals did not change the shape and chemical shift but gradually decreased the intensity, (b) eight new signals appeared in the region of 10.5–12.0 ppm, and six signals appeared in the region of 13.0–14.0 ppm increased the intensity as the molar ratio of **NCD** against **GG1** increased and (c) the signal changes saturated at the **NCD** and **GG1** ratio of 2:1. These results indicated that (i) the stoichiometry of **NCD** binding to **GG1** is 2:1 (**NCD**:**GG1**), (ii) the slow exchange of all imino protons between free **GG1** and **NCD**-**GG1** complex and (iii) the absence of signals regarding intermediates under the NMR measurement conditions. The 2:1 **NCD**:**GG1** binding stoichiometry observed by NMR titration experiments is fully consistent with the results obtained by MS and ITC analyses. The observed slow exchange of all imino protons indicated the strong binding of **NCD** to **GG1** (49). On the basis of these observations, two **NCD** molecules bind strongly to **GG1** with high cooperativity.

The direct evidence that **NCD** binding to the CGG/CGG triad is mediated by hydrogen bonding between naphthyridine moiety and guanine is the four strong NOE signals highlighted in red circles in Figure 3A, indicating the close proximity of the amide proton of NP and imino proton of the counterpart guanine (G6, G7, G17 and G18). These amide and imino protons were involved in the imino-imino sequential NOEs throughout the **NCD**-**GG1** complex, indicating that the four NP-G hydrogen-bonded pairs were stored in the helix. The incorporation of NP moieties into base stacks was suggested by the remarkable up-field shifts (around 4.8–5.0 ppm) of H1' protons of A4 and T15 (Supplementary Figure S2). These up-field shifts were likely due to the ring current effect of NP moieties. The invasive sequestration of G7 by NC4 and G18 by NC1 likely induced the flipping out of C16 and C5, respectively, as determined by chemical probing with hydroxylamine in our previous studies (18). The H6 protons of the C5 and C16 are significantly down-field shifted (8.01 ppm and 8.17 ppm, respectively. Supplementary Figure S4), supporting that these cytosines are not in the helix. As shown in Figure 5A, C5 and C16 have fewer NOE restraints than other residual nucleotides. In general, the more constraints of NOE, the better the convergence in the structural calculation. Therefore, the detailed conformation of the flipped-out cytosines in the **NCD**-**GG1** complex cannot be determined from these NMR experiments. In Figure 5C, the constraints of NOE used in the calculation are shown by the red line, and this figure also demonstrates that the flipped-out cytosines have not sufficient distance restraints.

**Figure 6.**
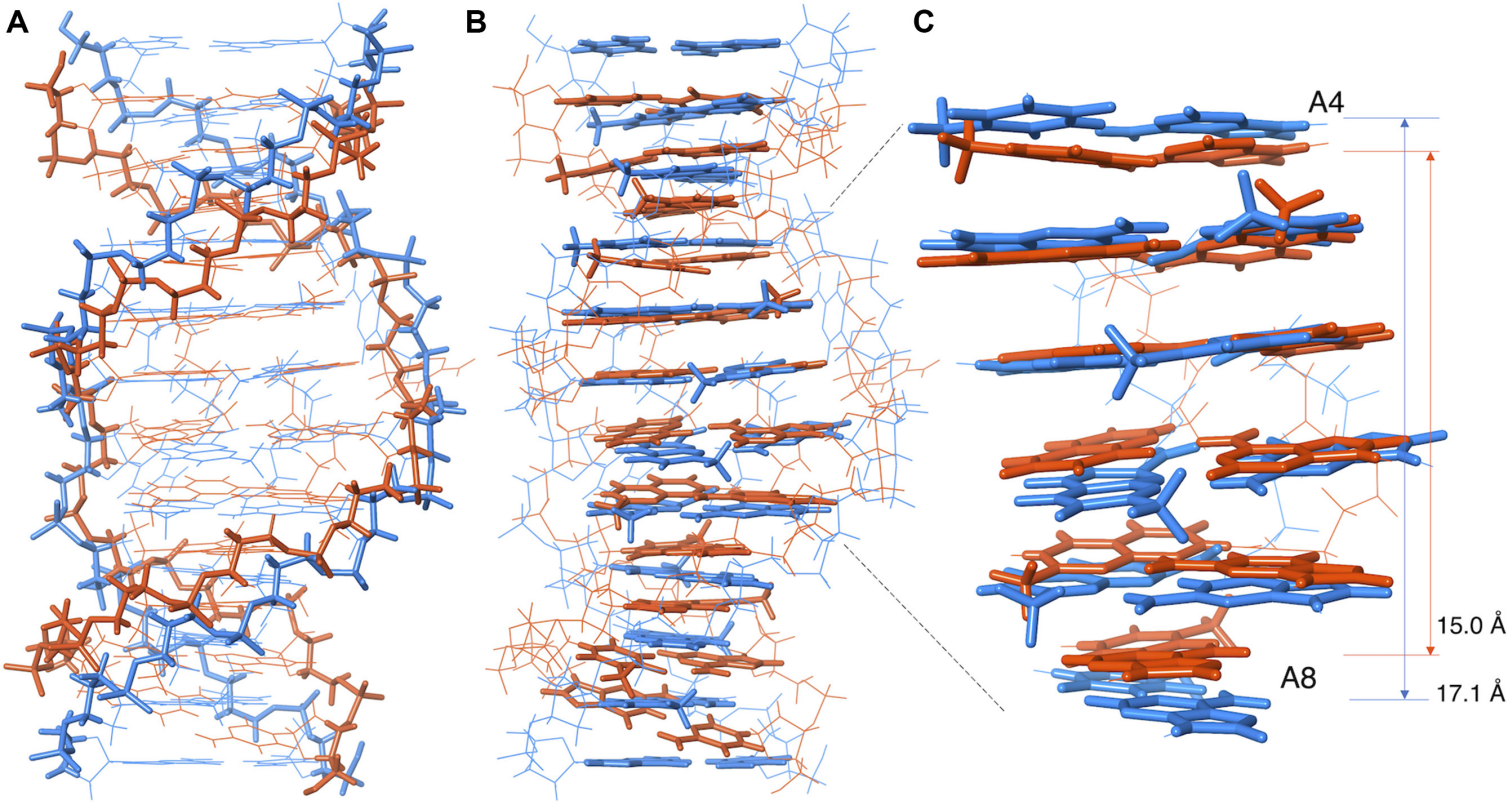
Superimpose of the state 1 structure of **NCD**-**GG1** (blue), and **NA**-**AA1** (orange) highlighted (**A**) the backbone and (**B**) base stacks. (**C**) Close up of the region from A4 to A8. The distances between two N1 atoms of A4 and A8 for **NCD**-**GG1** and **NA**-**AA1** were shown.

The NMR-determined structures of **NCD**-**GG1** were entirely consistent with the binding assay results showing (i) a 2:1 **NCD**:**GG1** binding stoichiometry and (ii) the flipping out of cytosine bases. Two **NCD** molecules bound to four guanines (G6, G7, G17 and G18) in the CGG/CGG triad by forming four pairs of NP-G hydrogen bonding. Two NP moieties of one **NCD** have a zigzag orientation in the complex to bind guanines in both strands (*cf*. Figure 1B). The linker moiety of **NCD** is located in the major groove, and the hydrogen-bonding surface of NP fits that of guanine in the anti-glycosidic conformation. These structural features are very similar to those of **NA**-**AA1**, but the overall structures of **NCD**-**GG1** are remarkably different from **NA**-**AA1**. Superimpose of the lowest energy state 1 structures of **NCD**-**GG1** (Figure 6A and B, blue) and **NA**-**AA1** (Figure 6A and B, orange) clearly showed that the **NCD**-**GG1** structure is about one-base pair longer than **NA**-**AA1**. The distance between two N1 atoms of A4 and A8 was 17.1 Å for **NCD**-**GG1**, thus 3.4 Å per step, whereas the distance was 15.0 Å for **NA**-**AA1** (3.0 Å per step), indicating that the stacking structure at the **NCD** and **NA** binding region is different from each other.

**Figure 7.**
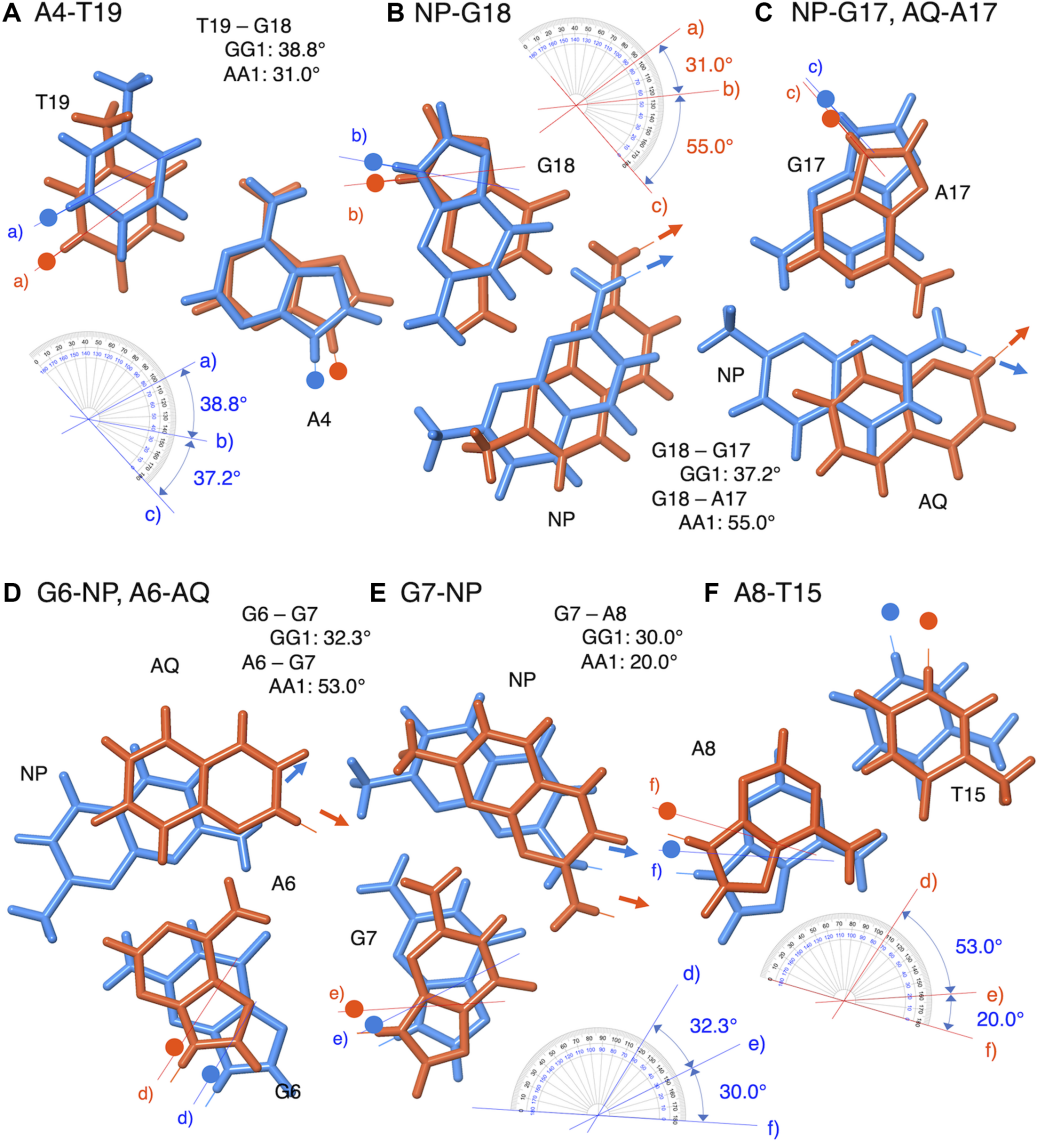
Superimpose of hydrogen bonding pairs in the ligand-bound region of state 1 of **NCD**-**GG1** (blue) and **NA**-**AA1** (orange) structures. Small circles represent the C1’ of deoxyribose and arrows indicate the direction of the connecting linker. (**A**) A4-T19, (**B**) NP-G18, (**C**) NP-G17 and AQ-A17, (**D**) G6-NP and A6-AQ, (**E**) G7-NP, (**F**) A8-T15. Twist angles were manually measured as the angle between two neighboring glycosidic bonds.

We compared precise positions of A4-T19, NP-G18, NP-G17 (AQ-A17), G6-NP (A6-AQ), G7-NP, and A8-T15 in the state 1 structures of **NCD**-**GG1** and **NA**-**AA1**, and found the following differences: (i) the direction of glycosidic bonds of G7 and G18 and (ii) the positions of NP and AQ opposite G6 (A6) and G17 (A17) (Figure 7). The twist angles determined by the dihedral angle between two glycosidic bonds of neighboring nucleotide bases showed that angles in the G18-A17 (55.0°) and A6-G7 (53.0°) steps in **NA**-**AA1** are significantly larger than those angles in **NCD**-**GG1** (G18-G17, 37.2°; G6-G7, 32.3°). Also, the angles in the G7-A8 (20.0°) and G18-T19 (31.0°) steps in **NA**-**AA1** are small to complement the large angles in the A6-G7 and A17-G18 steps, respectively. These data suggested that the G18 and G7 in **NA**-**AA1** bound to the NP moiety of **NA** were likely pulled toward the major groove, due to the short linker length connecting NP and AQ in **NA**. Regarding the NP and AQ position, NP is required to be inserted much deeper from the major groove toward the minor groove than AQ does due to the complementary hydrogen bond formation between NP and G (*cf*. Figure 7C and D). The linker moiety of **NCD** in the **NCD**-**GG1** located in the major groove would have more strain as the insertion of NP becomes deeper.

**Figure 8.**
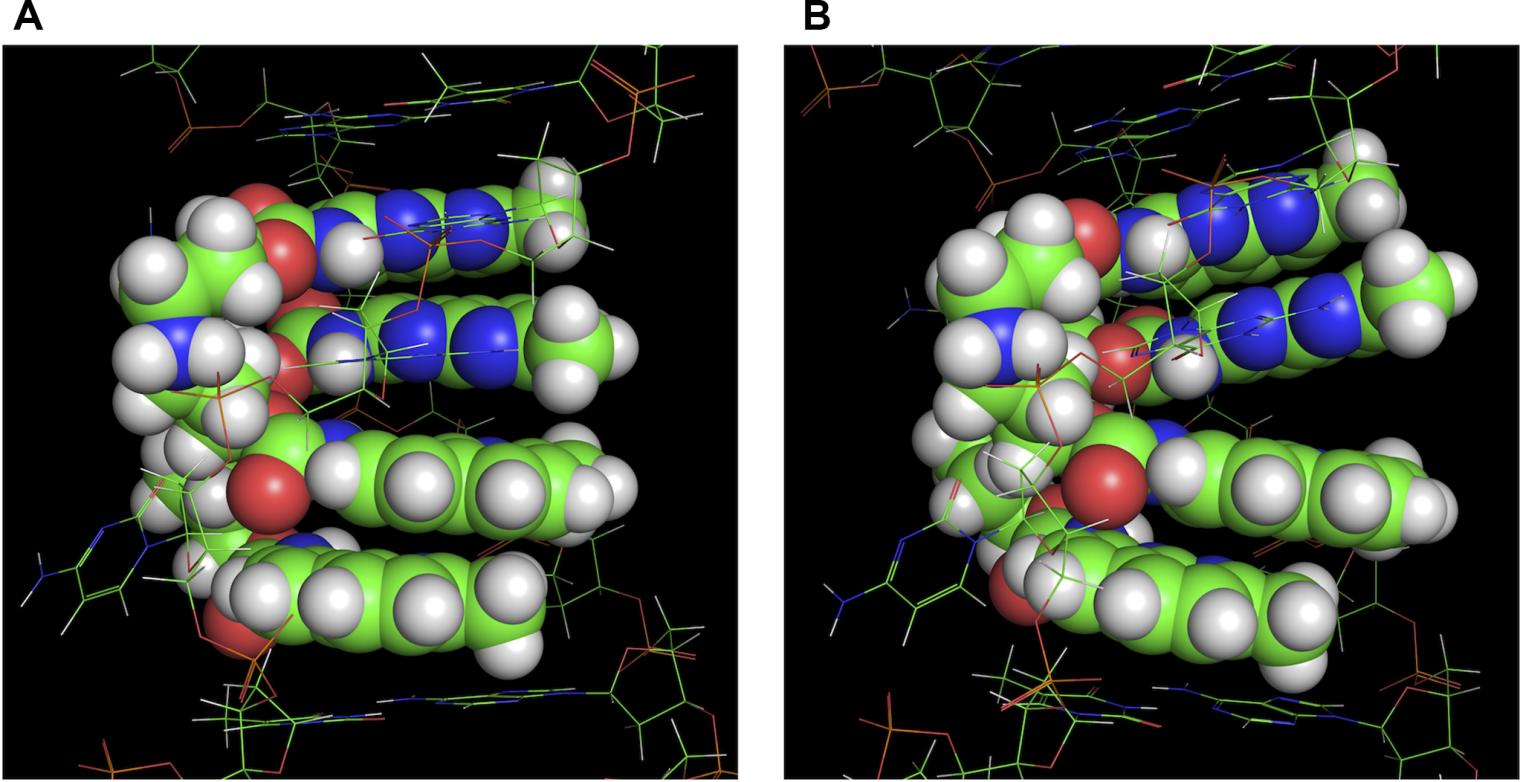
Two typical structures named ‘Stack’ and ‘Kink’ observed in the 30 lowest-energy structures. Two **NCD** molecules are shown as a sphere model (VDW scale is 0.8). DNA strands are shown by the line. The coloring of atoms is as follows; H = white, C = light green, N = blue, O = red. (**A**) The enlarged view of the state 1 structure, shown as a typical example of ‘Stack’ structure, where the base pairs are well stacked at the step between the G6-NP and NP-G17. (**B**) The enlarged view of state 2 structure, shown as an example of ‘Kink’ structure, where an apparent stacking defect is observed at the step between the G6-NP and NP-G17 pairs.

The 30 lowest-energy structures of **NCD**-**GG1** can be roughly classified into two groups, named ‘Stack’ and ‘Kink’ (Supplementary Figure S8). The representative structure of Stack (state 1) and Kink (state 2) were shown in Figure 8A. In structures of the Stack group, all base pairs seemed well stacked with a little distortion at the step between the G6-NP and NP-G17 producing B-form-like structures. In contrast, the Kink group structures showed an apparent stacking defect at the step between the G6-NP and NP-G17. Besides this step, base pairs and NP-G pairs produced well-stacked structures in both Stack and Kink groups. The correlation coefficient r between the experimental residual dipolar couplings (RDCs) (50,51) and the RDCs back-calculated from the structure (Supplementary Table S2) is a benchmark of the accuracy of the calculated structure. The average *r*-values for the Stack and Kink structures were both high as 0.80 and 0.83, respectively. The presence of two structure groups differentiated by the degree of stacking at G6-NP and NP-G17 pairs suggests that the **NCD**-**GG1** structures could dynamically fluctuate at that step. The linker's strain induced by deep insertion of NP in the Stack structures could be reduced in the Kink structures as the complex bends toward the major groove to make the distance of two NP moieties of **NCD** closer than that in the Stack structures. This suggests that the energy gain by stacking G6-NP and NP-G17 in the Stack structures would be balanced with the energy gain by releasing the strains in the Kink structure. Regarding **NA**-**AA1**, however, all 30 lowest-energy structures showed well-stacked structures throughout the duplex, including the steps between NP-G and AQ-A, and AQ-A and A-AQ (Supplementary Figure S9).

The molecular mechanism of **NA**-induced CAG repeats contraction *in vivo* is proposed to involve the escape of the repair process of **NA**-bound CAG hairpin produced during the transcription. These findings have implicated that repeat contraction on other repeats such as CGG, CTG, and CCG might be conceivable if small molecules, like **NA**, binding to these repeat sequences are available. We reported **NCD** as a molecule binding to CGG repeat DNA, but the **NCD**-bound structure was left undetermined. NMR structure of the **NCD**-CGG/CGG triad shared several structural characteristics with that of the **NA**-CAG/CAG triad. However, the dynamic equilibrium was suggested only for the **NCD**-CGG/CGG triad. Since most repair enzymes sense the DNA damage sites by the local deformability (52,53), the **NCD**-CGG/CGG structures having dynamic equilibrium between two states might be more susceptible to the binding of repair enzymes, eventually leading to the replacement of **NCD** with repair enzymes. The effect of the observed structural dynamics on contraction efficiency will be the subject of our next research.

## CONCLUSIONS

Similarities in the binding properties, i.e. 2:1 ligand:DNA binding stoichiometry, cytosine flipping out, and significant increase of thermodynamic stability between the **NA**-binding to the CAG/CAG motif and NCD-binding to the CGG/CGG motif in dsDNA were discussed by the NMR structure determination of the **NCD**-**GG1** complex. The determined structure of the **NCD**-**GG1** complex is fully consistent with the binding properties and confirmed the hydrogen bonding of naphthyridine moieties of **NCD** with four guanines in the CGG/CGG motif. The hydrogen-bonded pairs of naphthyridine-guanine were stored in the helical structure of dsDNA. Although the hydrogen-bonding interactions and resultant cytosine flipping out were confirmed to be similar between **NCD**-**GG1** and **NA**-**AA1**, there were significant differences in the local structures at the ligand-bound region. In the **NA**-**AA1** complex, both naphthyridine and azaquinolone were biased toward the major groove due to the short linker length of the **NA**, whereas in the **NCD**-**GG1** complex, the naphthyridine–guanine hydrogen bond pairs are pulled into the minor groove side, suggesting that the linker moiety of **NCD** has more strain than that of **NA** linker had. The NMR analysis suggested that the linker structure of **NCD** still has some room for optimization to gain the stacking energy between two NP-G pairs and to release a strain in the linker. We are currently working on the structure binding studies of **NCD** derivatives. In these studies, the **NCD**-**GG1** structure provides the starting point for the quantum calculations and molecular dynamic simulations (Supplementary Figure S10), which may eventually lead to the design of molecules with higher affinity to the CGG/CGG triad, and hopefully, the CGG repeat DNA. Our recent studies on **NA** binding to the CAG repeat DNA *in vivo* suggested the **NA**-bound CAG repeat hairpin could be escaped from the repair mechanism, eventually leading to the repeat contraction. Thus, the stronger binding of **NCD** derivatives to the CGG repeat, the more chance of escape from the repair system is likely to be in the scope.

## DATA AVAILABILITY

accession code of the 3D structure and the chemical shift of the NCD-DNA complex: PDB ID 7YVW, BMRB ID 36507.

## Supplementary Material

gkac740_Supplemental_File
