# Supplementary material for: NMR determination of the 2:1 binding complex of naphthyridine carbamate dimer (NCD) and CGG/CGG triad in double-stranded DNA
Source: Nucleic Acids Res. 2022 Sep 12;50(17):9621–31. doi: 10.1093/nar/gkac740 (PMC9508812; doi:10.1093/nar/gkac740)
Supplement: gkac740_Supplemental_File [file gkac740_supplemental_file.docx]

Supporting Information for

**NMR determination of the 2:1 binding complex of naphthyridine carbamate dimer (NCD) and CGG/CGG triad in double-strand DNA**

Takeshi Yamada^1^, Kyoko Furuita^2^, Shuhei Sakurabayashi^1,2^, Makoto Nomura^3^, Chojiro Kojima^2,4^, and Kazuhiko Nakatani^1, *^

^1^ Department of Regulatory Bioorganic Chemistry, SANKEN, Osaka University, 8-1 Mihogaoka, Ibaraki 567-0047, Japan

^2^Institute for Protein Research, Osaka University, 3-2 Yamadaoka, Suita, Osaka 565-0871, Japan

^3^Graduate School of Biological Sciences, Nara Institute of Science and Technology, 8916-5 Takayama, Ikoma 630-0192, Japan

^4^Graduate School of Engineering Science, Yokohama National University, 79-5 Tokiwadai, Hodogaya-ku, Yokohama 240-8501, Japan

**Figure S1**. Binding analyses of **NCD** to **GG1**. **(A)** Thermal melting profiles of **GG1** (5 µM) in sodium cacodylate buffer (10 mM, pH 7.0) containing NaCl (100 mM) in the absence (black circle) and presence of **NCD** (20 µM, red circle). (**B)** Circular dichroism (CD) spectrum of **GG1** (5 µM) measured in sodium cacodylate buffer (10 mM, pH 7.0) containing NaCl (100 mM) at room temperature in the absence (black circle) and presence of **NCD** (20 µM: red circle). **(C)** Cold spray ionization time-of-flight (CSI-TOF) MS profiles of the mixed solution of H_2_O/MeOH (1:1, v/v) containing DNA (5’-CTAA CGG AATG TTTT CATT CGG TTAG-3’; 5 μM) and ammonium acetate (100 mM) in the absence (above) and presence (below) of **NCD** (100 µM). **(D)** The result of isothermal titration calorimetry (ITC) Above: the titration profile of sodium cacodylate buffer (10 mM, pH 7.0) ) solution containing **NCD** (50 μM) and NaCl (100 mM) to the sodium cacodylate buffer (10 mM, pH 7.0) solution containing **GG1** (2.5 μM) and NaCl (100 mM) at 25 °C. Below: the integrated heat plots obtained from the above profile with the best fit curve to the one-set-of-sites model.


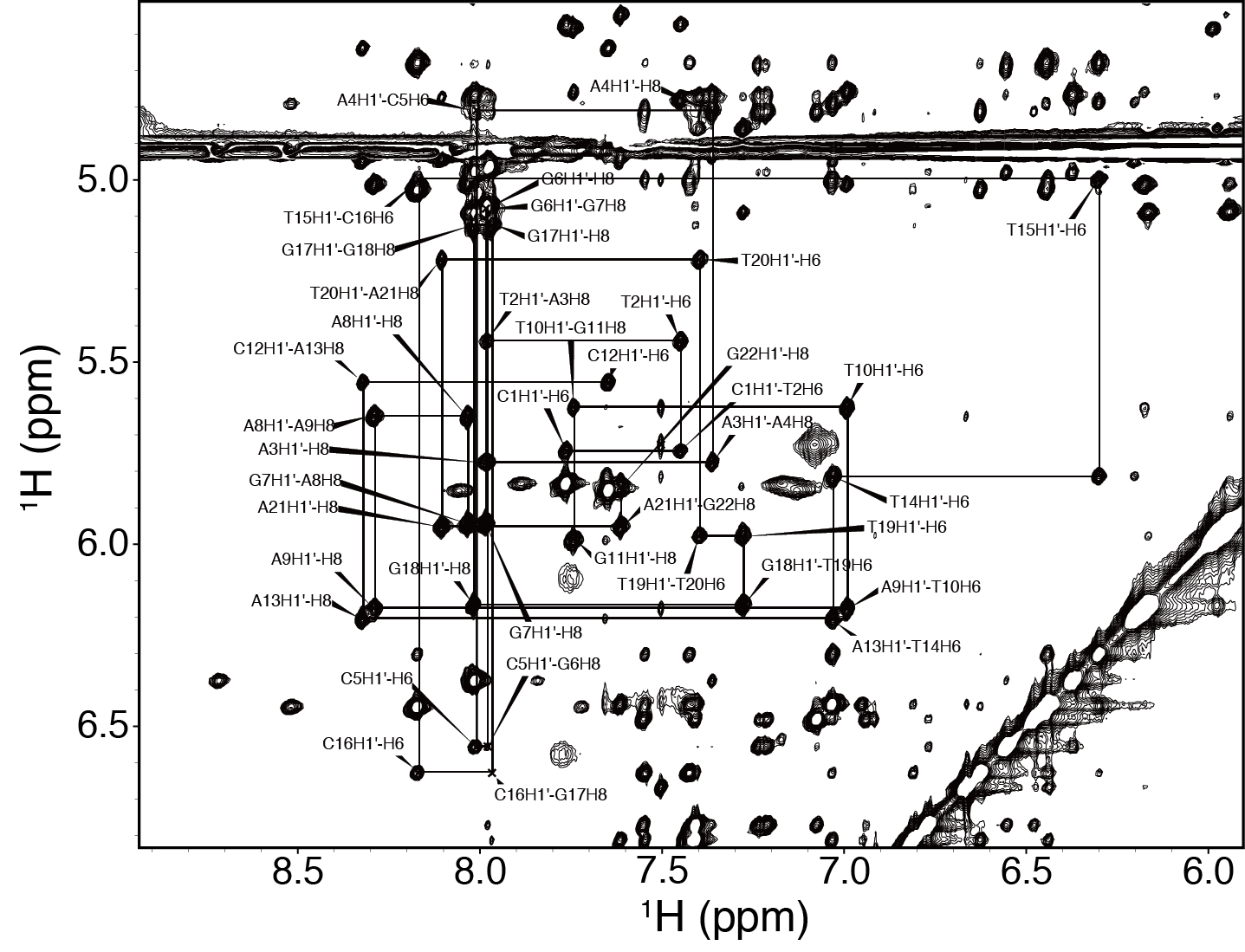


**Figure S2.** Expansion of ^1^H-^1^H NOESY spectrum of **NCD**-**GG1**. The sequential cross peaks between pyrimidine-H6 or purine-H8 and deoxyribose-H1’ are highlighted by lines.


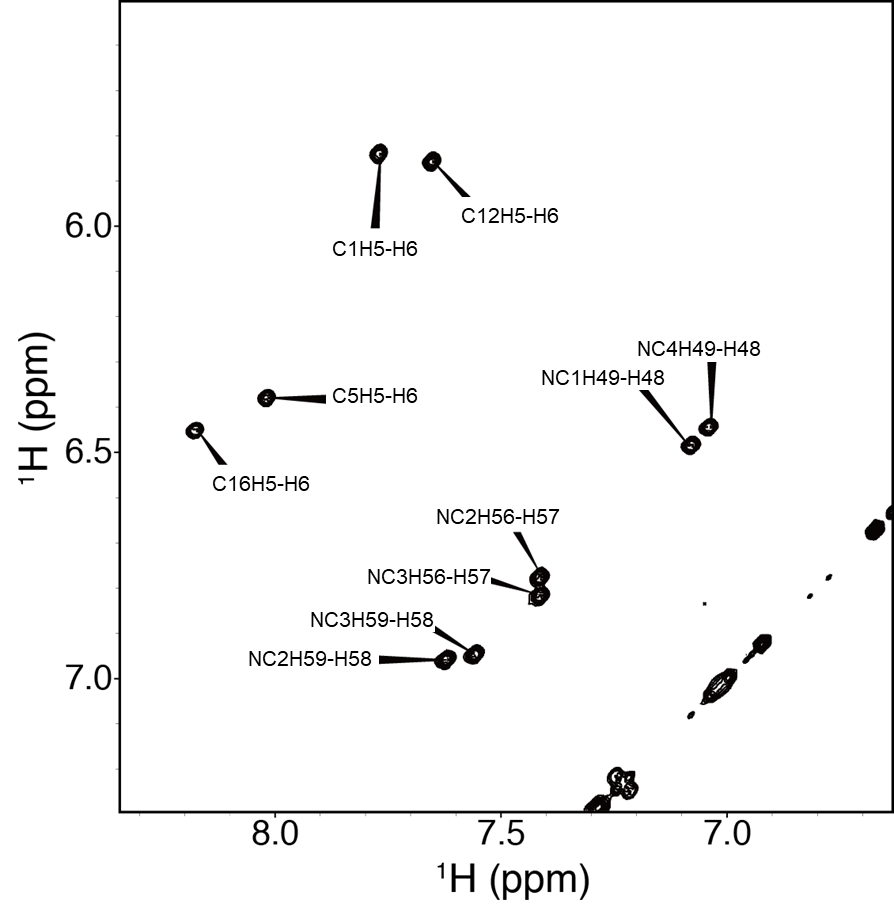


**Figure S3.** The ^1^H-^1^H TOCSY spectrum of **NCD**-**GG1**. Peaks of cytosine bases and NP moieties of **NCD** molecules are shown. The numbering of **NP** moieties of **NCD** is shown in Fig 1D.


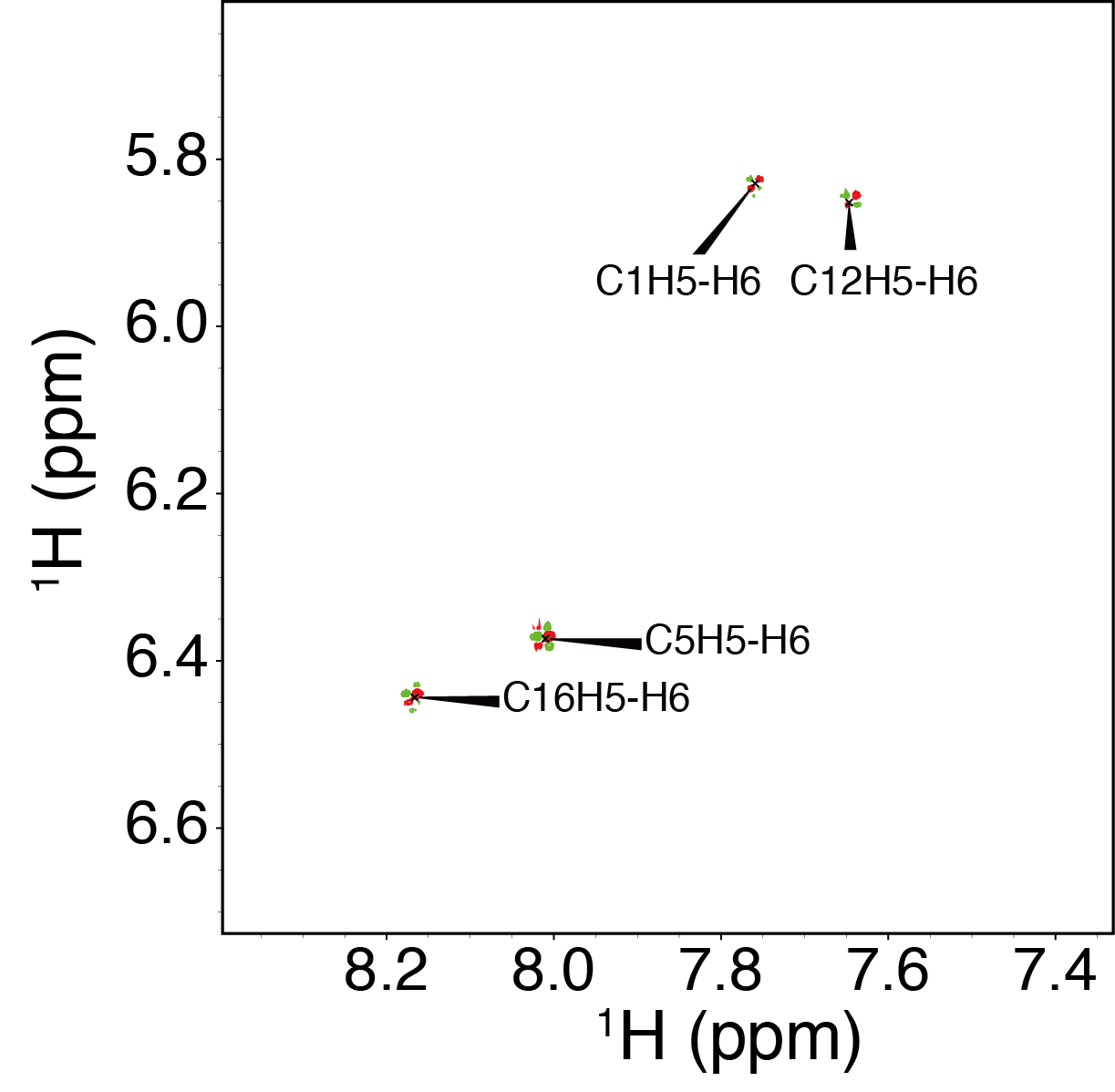


**Figure S4.** The DQF-COSY spectrum of **NCD**-**GG1**. Cross peaks between cytosine H5 and H6 are shown.


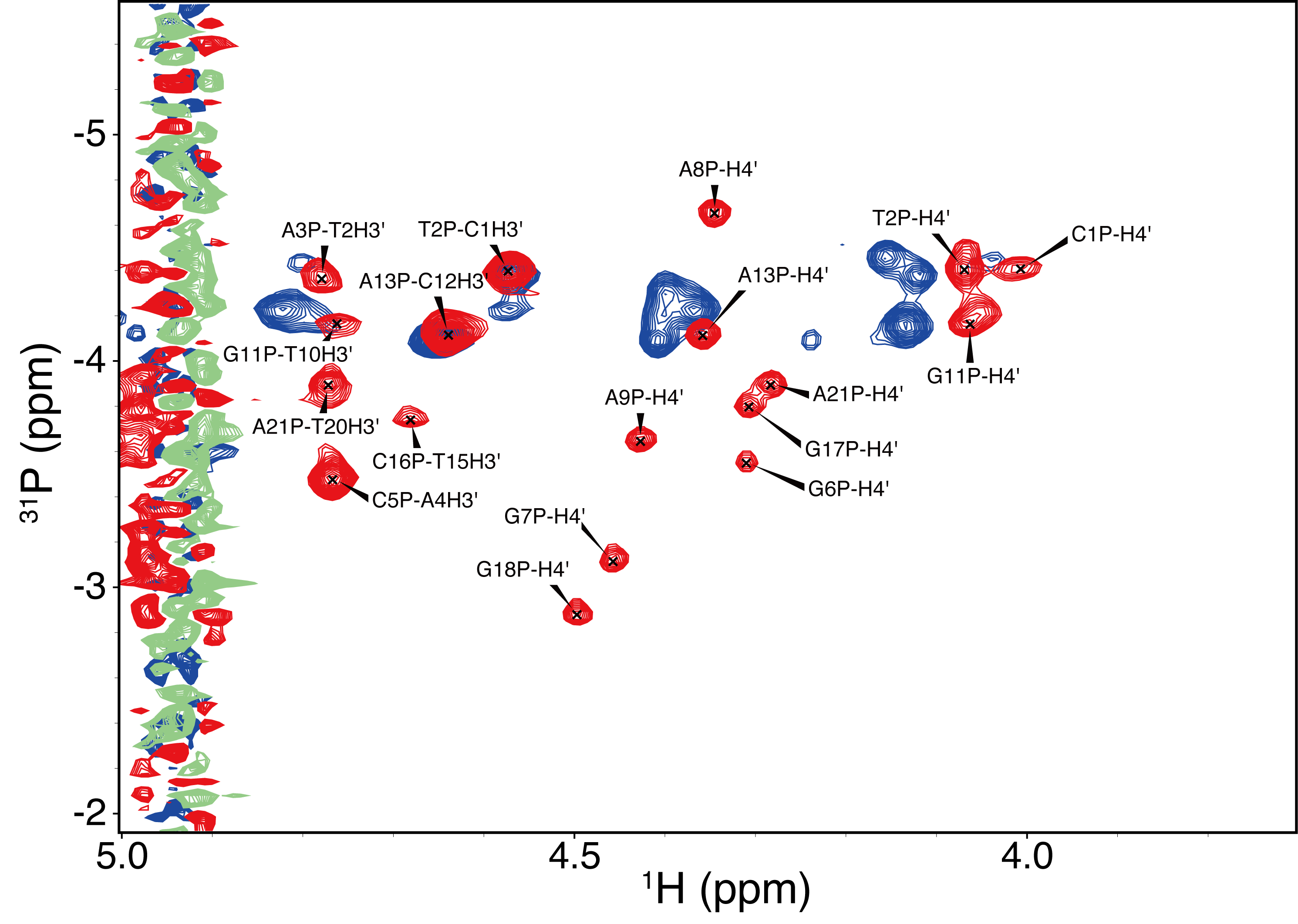


**Figure S5**. An overlay of ^1^H-^31^P HSQC spectra of **GG1** (blue) and **NCD**-**GG1** (red). The peaks of **NCD**-**GG1** are labeled.

**
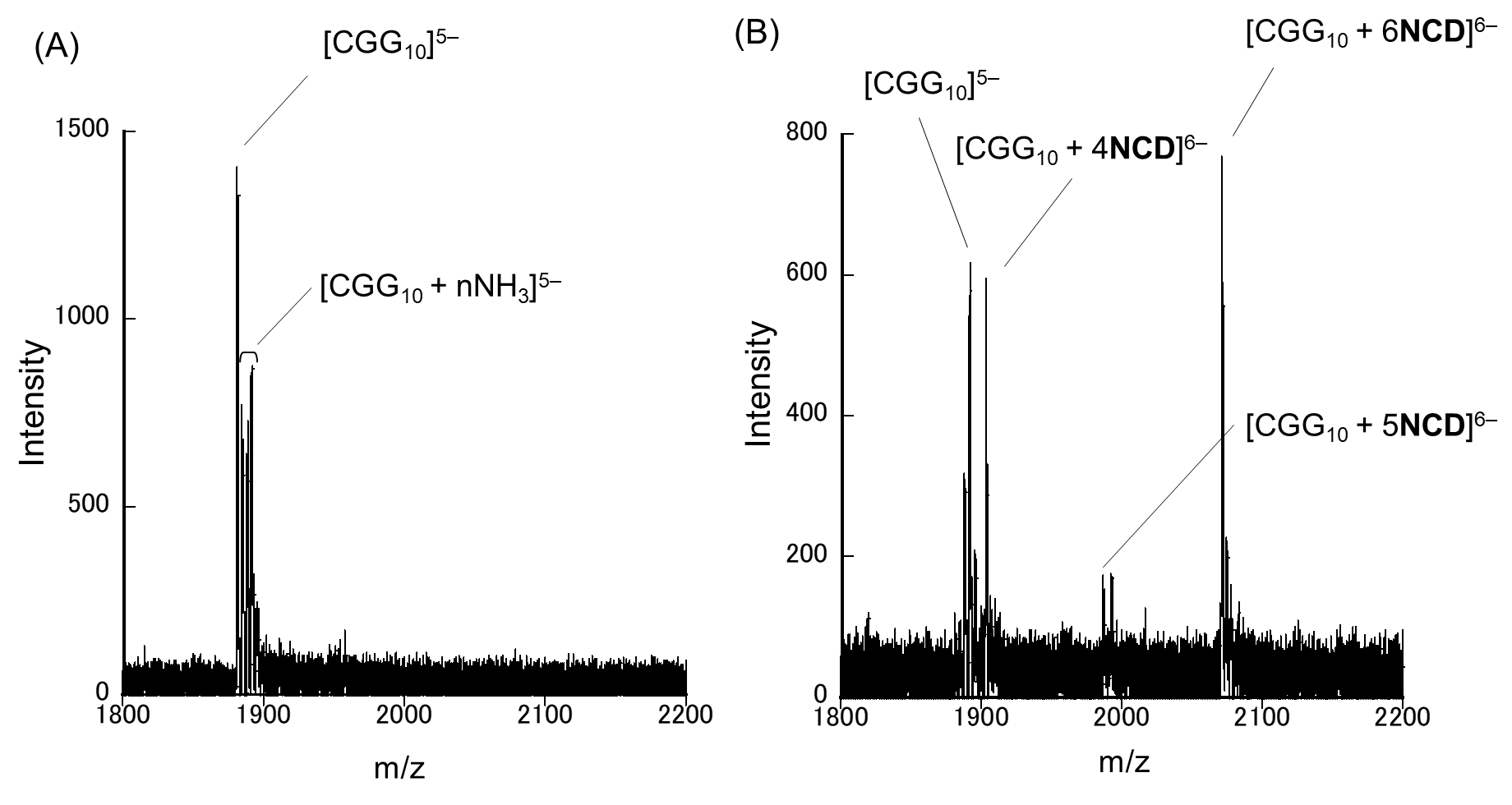
**

**Figure S6**. CSI-TOF mass spectra of d(CGG)_10_ in the absence and presence of **NCD**. Samples contain 10 µM of DNA in 50% aqueous methanol and 100 mM ammonium acetate. For clarity, ions in the range of m/z from 1800 to 2200 were shown. The sample solution was cooled at –10 ºC during the injection. (a) d(CGG)_10_ only, and (b) d(CGG)_10_ with 50 µM **NCD**.

**
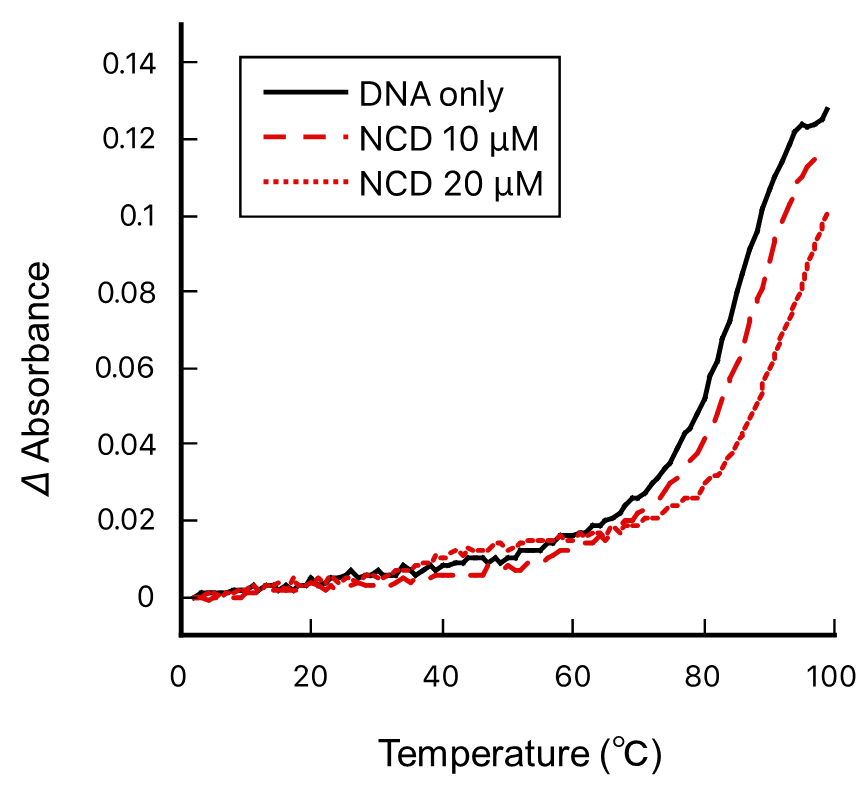
**

**Figure S7**. Thermal melting profiles of d(CGG)_10_ (2 µM) in sodium cacodylate buffer (10 mM, pH 7.0) containing NaCl (100 mM) in the absence (black line) and presence of **NCD** (10, and 20 µM).

**Table S1**. Structural statistics of **NCD**-**GG1**.

| Total number of distance constraints | 358 |
| --- | --- |
| Intra residue | 143 |
| Inter residue | 105 |
| Intermolecular (**NCD**-DNA) | 42 |
| H-bond | 68 |
| Dihedral constraints | 180 |
| CNS energy terms (kcal/mol) |  |
| E_bond_ | 32.24 ± 0.86 |
| E_angles_ | 134.24 ± 5.85 |
| E_imp_ | 45.89 ± 1.60 |
| E_vdw_ | -242.22 ± 8.18 |
| R.m.s. deviations to mean structure of the 30 calculated structures |  |
| All atom (Å) | 1.21 ± 0.36 |
| Back bone (Å) | 1.00 ± 0.42 |
| All atom (2-4,6-10,13-15,17-21,NCD1,NCD2) (Å) | 0.89 ± 0.31 |
| Backbone (2-4,6-10,13-15,17-21) (Å) | 0.90 ± 0.42 |

**Table S2.** The classification and r values of the lowest 30 structures of **NCD**-**GG1**.

| State No. | Group | R values |
| --- | --- | --- |
| 1 | Stack | 0.843 |
| 2 | Kink | 0.871 |
| 3 | Kink | 0.881 |
| 4 | Stack | 0.789 |
| 5 | Stack | 0.767 |
| 6 | Kink | 0.809 |
| 7 | Kink | 0.827 |
| 8 | Kink | 0.861 |
| 9 | Stack | 0.895 |
| 10 | Kink | 0.856 |
| 11 | Kink | 0.766 |
| 12 | Kink | 0.895 |
| 13 | Kink | 0.847 |
| 14 | Kink | 0.857 |
| 15 | Kink | 0.868 |
| 16 | Kink | 0.860 |
| 17 | Stack | 0.804 |
| 18 | Kink | 0.825 |
| 19 | Kink | 0.746 |
| 20 | Kink | 0.862 |
| 21 | Stack | 0.748 |
| 22 | Kink | 0.854 |
| 23 | Kink | 0.90 |
| 24 | Kink | 0.884 |
| 25 | Kink | 0.82 |
| 26 | Kink | 0.807 |
| 27 | Kink | 0.817 |
| 28 | Kink | 0.845 |
| 29 | Kink | 0.809 |
| 30 | Kink | 0.834 |

**Figure S8.** The 30 lowest-energy structures showing the stacking of NP-G hydrogen-bonded pairs with neighboring base pairs in **NCD**-**GG1**. A4-T19, NP-G18, NP-G17, G6-NP, G7-NP, and A8-T16 were shown as a sphere model (VDW scale is 1.0). Flipped-out C5 and C16 were shown as a stick model. The numbers displayed at the bottom of each structure are the correlation coefficient (r) of RDCs of the experiments and those back-calculated.

**Figure S9.** The 30 lowest-energy structures showing the stacking of NP-G and AQ-A hydrogen-bonded pairs with neighboring base pairs in **NA**-**AA1**. A4-T19, NP-G18, AQ-A17, A6-AQ, G7-NP, and A8-T16 were shown as a sphere model (VDW scale is 1.0). Flipped-out C5 and C16 were shown as a stick model.


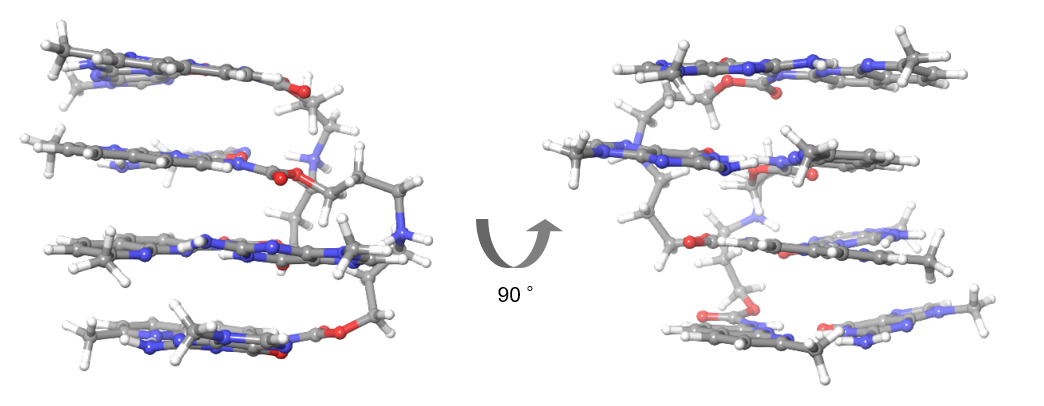


**Figure S10.** A view of geometry of **NCD**-**GG1** ligand-bound region optimized by DFT calculation.

# Methods

### Melting temperature (*T*_m_) measurements

*T*_m_ profiles of the sodium cacodylate buffer (10 mM, pH 7.0) solutions of **GG1** (5’-(TCAA CGG TTGA)-3’/3’-(AGTT GGC AACT)-5’: 5 µM) containing NaCl (100 mM), in the absence or the presence of **NCD** (20 µM), were recorded by ﻿a UV-Vis spectrometer (UV-2550, SHIMADZU) equipped with a temperature controller (TMSPC-8, SHIMADZU). *T*_m_ profiles of the d(CGG)_10­_ in the absence or presence of **NCD** (10 and 20 µM) is also analyzed under the same conditions as mentioned above. Experiments were performed at least three times on the same condition to obtain the mean value and the standard deviation.

### Circular dichroism (CD) spectrum measurements

CD measurements of the sodium cacodylate buffer (10 mM, pH 7.0) solutions of **GG1** (5’-(TCAA CGG TTGA)-3’/3’-(AGTT GGC AACT)-5’: 5 µM) containing NaCl (100 mM), in the absence or the presence of **NCD** (20 µM), were recorded by ﻿a CD spectropolarimeter (J-725, JASCO).

### CSI-TOF MS spectrum measurements

CSI-TOF MS spectrum measurements of 10 µM d(CGG)­_10_ in 50% aqueous methanol and 100 mM ammonium acetate, in the absence or the presence of **NCD** (50 µM), were recorded by with JEOL JMS-T100LP AccuTOF LC-plus 4G mass spectrometer in negative mode. Spray temperature was fixed at –10 °C. CSI-TOF MS spectrum measurements of 5 µM **GG1** in 50% aqueous methanol and 100 mM ammonium acetate, in the absence or the presence of **NCD** (100 µM), were recorded under the same conditions as mentioned above.

### Isothermal titration calorimetry (ITC).

ITC measurements were performed on a calorimeter (MicroCal iTC200, Malvern Panalytical), and data analysis was carried out with Origin 7.0 software. A 600 µL solution of **GG1** (2.5 µM) in sodium cacodylate buffer (10 mM, pH 7.0) containing NaCl (100 mM) was prepared, and then a part of the solution was put in the cell of the calorimeter. The temperature inside the cell was kept at 25 °C during the experiment. Next, a 200 µL sodium cacodylate buffer (10 mM, pH 7.0) solution of **NCD** (50 µM) containing NaCl (100 mM) was prepared as a titrant, and then a part of the solution was put in the 40 µL syringe of the calorimeter. The titration experiment was performed by the 19 times injections of the titrant (0.4 µL for the first injection and 2 µL for the other injections) from the syringe into the sample cell at 25 °C (stirred at 750 rpm). The initial time prior to the first injection was 60 seconds. The duration for each injection was 4 seconds, and the interval between two nearest injections was 150 seconds. In addition, the titrant was injected to the cell containing H_2_O (200 µL) in the same manner to obtain the data for the heat of dilution of the titrant. Prior to fitting analysis, the data point corresponding to the first injection was removed, and the heat data was corrected by subtracting the heat of dilution. Binding isotherms were fitted to the Origin models by least-squares analysis. Experiments were performed at least three times on the same condition to obtain the mean value and the standard deviation.

### DFT calculation

Structure optimization of the ligand-bound region of **NCD**-**GG1** was performed by Gaussian 16 using parameter of ωB97XD/6-31+G(d). The ligand-bound region of **NCD**-**GG1** was used as the initial structure, with the phosphate backbone removed and the deoxyribose moieties replaced with methyl groups.
